# A multicenter analysis of trends in resistance in urinary Enterobacterales isolates from ambulatory patients in the United States: 2011–2020

**DOI:** 10.1186/s12879-022-07167-y

**Published:** 2022-02-28

**Authors:** Michael W. Dunne, Steven I. Aronin, Kalvin C. Yu, Janet A. Watts, Vikas Gupta

**Affiliations:** 1 Bill & Melinda Gates Medical Research Institute, Cambridge, MA USA; 2 Iterum Therapeutics, Old Saybrook, Connecticut, USA; 3 Becton, Dickinson and Company, 1 Becton Drive, Franklin Lakes, NJ 07417 USA

**Keywords:** Enterobacteriaceae, Urinary tract infection, Antimicrobial resistance, Fluoroquinolones, Extended-spectrum beta-lactamase

## Abstract

**Background:**

Urinary tract infections (UTIs), which are usually caused by bacteria in the Enterobacterales family, are a common reason for outpatient visits. Appropriate empiric therapy for UTIs requires an understanding of antibiotic resistance in the community. In this nationwide study, we examined trends in antibiotic resistance in urinary Enterobacterales isolates from ambulatory patients in the United States (US).

**Methods:**

We analyzed the antimicrobial susceptibility profiles (extended-spectrum beta-lactamase [ESBL]-producing phenotype and not susceptible [NS] to beta-lactams, trimethoprim/sulfamethoxazole [TMP/SMX], fluoroquinolones [FQ], or nitrofurantoin [NFT]) of 30-day non-duplicate Enterobacterales isolates from urine cultures tested at ambulatory centers in the BD Insights Research Database (2011–2020). The outcome of interest was the percentage of resistant isolates by pathogen and year. Multi-variable generalized estimating equation models were used to assess trends in resistance over time and by additional covariates.

**Results:**

A total of 338 US facilities provided data for > 2.2 million urinary Enterobacterales isolates during the 10-year study. Almost three-quarters (72.8%) of Enterobacterales isolates were *Escherichia coli*. Overall unadjusted resistance rates in Enterobacterales isolates were 57.5%, 23.1%, 20.6%, and 20.2% for beta-lactams, TMP/SMX, FQ, and NFT, respectively, and 6.9% had an ESBL-producing phenotype. Resistance to two or more antibiotic classes occurred in 16.4% of isolates and 5.5% were resistant to three or more classes. Among isolates with an ESBL-producing phenotype, 70.1%, 59.9%, and 33.5% were NS to FQ, TMP/SMX, and NFT, respectively. In multivariable models, ESBL-producing and NFT NS Enterobacterales isolates increased significantly (both P < 0.001), while other categories of resistance decreased. High rates (≥ 50%) of beta-lactam and NFT resistance were observed in *Klebsiella* isolates and in non-*E. coli*, non-*Klebsiella* Enterobacterales isolates.

**Conclusions:**

Antimicrobial resistance was common in urinary Enterobacterales isolates. Isolates with an ESBL-producing phenotype increased by about 30% between 2011 and 2020, and significant increases were also observed in NFT NS Enterobacterales isolates. Resistance rates for all four antibiotic classes were higher than thresholds recommended for use as empiric therapy. Non-*E. coli* Enterobacterales isolates showed high levels of resistance to commonly used empiric antibiotics, including NFT. These data may help inform empiric therapy choices for outpatients with UTIs.

**Supplementary Information:**

The online version contains supplementary material available at 10.1186/s12879-022-07167-y.

## Background

Urinary tract infections (UTIs) are a common reason for outpatient and emergency department visits. Between 2006 and 2010, UTI visits accounted for an estimated 40.9 million ambulatory visits in the United States [1], and the rate of outpatient UTIs is increasing [2]. Most community-onset uncomplicated UTIs (uUTIs) are caused by members of the Enterobacterales family, including *Escherichia coli*, *Klebsiella pneumoniae*, and *Proteus mirabilis* [3]. Increasing rates of antimicrobial resistance in ambulatory UTI Enterobacterales isolates have obscured disease management [4–7]. Antibiotic-resistant UTIs are associated with high rates of discordance between antibiotic selection and isolate susceptibility and with treatment failure [8–11]. Furthermore, antimicrobial resistance has been proposed as a key driver for the dramatic increase in US hospitalizations for UTIs due to the need for treatment with intravenous antimicrobials [12].

Infectious Disease Society of America (IDSA) guidelines from 2010 recommend several therapeutic options for empiric treatment of uUTIs (cystitis), including nitrofurantoin (NFT), trimethoprim/sulfamethoxazole (TMP/SMX), and fosfomycin; fluoroquinolones (FQ) and beta-lactams are listed as additional options if the recommended antimicrobials cannot be used or are not effective [3]. The IDSA guidelines also specify that TMP/SMX should be avoided in treatment of acute cystitis if local resistance rates are > 20%, and FQ should be avoided in treatment of acute pyelonephritis if local resistance rates are > 10%. Of note, the US Food and Drug Administration (FDA) subsequently issued a drug safety announcement indicating that FQ should not be used for uUTIs because of the risk of serious side effects [13]. As a result, there are now limited preferred agents for uUTIs, and antimicrobial resistance further diminishes their utility.

Appropriate empiric management of outpatient UTIs requires an understanding of current antimicrobial susceptibility profiles for key pathogens circulating in the community. The objective of the study was to examine trends in antibiotic resistance in Enterobacterales isolated from urine cultures tested in the ambulatory setting.

## Methods

### Study design

These analyses are derived from a retrospective cohort study of antimicrobial susceptibility of specified non-duplicate (first isolate of a species in 30 days) Enterobacterales urine isolates from adult male and female patients (age ≥ 18 years) collected in the ambulatory setting (e.g., non-inpatient with no subsequent admission in the next day) during the time period spanning the first quarter (Q1) of 2011 to the last quarter (Q4) of 2020. Our analyses were based solely on culture results; patients were not required to have symptoms or a UTI diagnosis to be included in the study. Reporting institutions consisted of ambulatory sites affiliated with US hospitals in the BD Insights Research Database (Becton, Dickinson and Company, Franklin Lakes, NJ), which provides geographical representation across the US and includes small and large hospitals in urban and rural areas [14–16].

Pathogen identification and susceptibility results were based on facility reports from healthcare facilities in the BD Insights Research Database using commercial panels and local laboratory breakpoints. Central laboratory testing was not performed. We evaluated five categories of antimicrobial resistance in Enterobacterales urine isolates using the following definitions:

1. Organisms with an extended-spectrum beta-lactamase (ESBL)-producing phenotype: *E. coli*, *K. pneumoniae*, *Klebsiella oxytoca*, and *P. mirabilis* urine isolates confirmed as ESBL-positive per commercial panels or based on a result of intermediate (I) or resistant (R) to antimicrobial susceptibility tests with extended-spectrum cephalosporins (ceftriaxone, cefotaxime, ceftazidime, or cefepime; ESC4).
2. Beta-lactam not susceptible (NS) organisms: Isolates with an ESBL-producing phenotype as defined above, or *E. coli, K. pneumoniae, K. oxytoca, Klebsiella aerogenes, P. mirabilis, Enterobacter cloacae, Serratia marcescens, Citrobacter freundii, Providencia stuartii* and *Morganella morganii* isolates that tested I or R to aminopenicillins (including ampicillin/sulbactam), 1st/2nd/3rd/4th-generation cephalosporins, piperacillin/tazobactam, or carbapenems.
3. TMP/SMX NS organisms: Enterobacterales urine isolates testing I or R to TMP/SMX.
4. FQ NS organisms: Enterobacterales urine isolates testing I or R to ciprofloxacin, levofloxacin, or moxifloxacin.
5. NFT NS organisms: Enterobacterales urine isolates testing I or R to NFT.

### Outcomes

For each of the five categories of resistance defined above, we evaluated the percent of resistance (number of resistant isolates per total isolates tested) overall, by specific pathogen, by year, and by treatment facility characteristics.

### Statistical analysis

Descriptive statistics of percent of resistant isolates over time were presented by cross-tabulation. The covariates considered in the multi-variable modeling analysis included hospital bed size (grouped to three categories: < 100, 100–300, and > 300), urban/rural status, teaching status, and geographic region (based on US census regions). Generalized estimating equation GEE models with autoregressive variance–covariance structure and with hospitals as random effect were used to assess the trends of resistance over time (years) and evaluate the effects of season as determined by quarterly data and other covariates on resistance. All statistical analyses were conducted using R V 4.0.3 (R Core Team 2020) and the R geepack package. *P* values < 0.05 were considered statistically significant.

## Results

A total of 338 facilities provided data during the 10-year study period (Additional file 1: Table S1). About two-thirds of the facilities (65.7%) were classified as urban, 67.8% were affiliated with non-teaching hospitals, and 24.9% were affiliated with hospitals with > 300 beds. Geographically, the largest concentrations of facilities were in the West South Central (18.9%) and Middle Atlantic (16.9%) region.

### Resistance by drug class

Over the 10-year period, more than 2 million Enterobacterales urinary isolates were evaluated for an ESBL-producing phenotype and more than 2.2 million isolates were evaluated for the other resistance profiles. Of the 2,228,515 urinary isolates evaluated for beta-lactam, TMP/SMX, FQ, and NFT resistance, the most common bacteria were *E. coli* (1,623,448 [72.8%]), *K. pneumoniae* (301,628 [13.5%]) and *P. mirabilis* (139,204 [6.2%]) (Table 1). Descriptive statistics showed that the highest rate of antibiotic resistance in Enterobacterales urinary isolates over the 10-year period was to beta-lactams (57.5%), followed by TMP/SMX (23.1%), FQ (20.6%), NFT (20.2%), and ESBL-producing phenotype (6.9%) (Table 1). Quarterly mean rates of resistance were similar (Additional file 1: Table S2).

**Table 1:** Descriptive statistics of antimicrobial resistance in ambulatory-onset urine Enterobacterales isolates (2011–2020)

Organism	Antimicrobial resistance														
	ESBL phenotype			Beta-lactam			Trimethoprim/sulfamethoxazole			Fluoroquinolones			Nitrofurantoin		
	Tested	NS	% NS	Tested	NS	% NS	Tested	NS	% NS	Tested	NS	% NS	Tested	NS	% NS
All	2,095,447	145,448	6.9	2,228,515	1,280,780	57.5	2,228,515	513,945	23.1	2,228,515	459,339	20.6	2,228,515	450,818	20.2
* E. coli*	1,623,448	116,843	7.2	1,623,448	817,083	50.3	1,623,448	422,699	26.0	1,623,448	376,459	23.2	1,623,448	64,194	4.0
* K. pneumoniae*	301,628	17,359	5.8	301,628	274,284	90.9	301,628	33,231	11.0	301,628	18,003	6.0	301,628	174,453	57.8
* P. mirabilis*	139,204	8991	6.5	139,204	38,423	27.6	139,204	36,105	25.9	139,204	43,767	31.4	139,204	124,276	89.3
* E. cloacae*				39,007	35,090	90.0	39,007	5442	14.0	39,007	3110	8.0	39,007	25,143	64.5
* K. oxytoca*	31,167	2255	7.2	31,167	29,636	95.1	31,167	2939	9.4	31,167	1715	5.5	31,167	5010	16.1
* K. aerogenes*				30,234	26,770	88.5	30,234	836	2.8	30,234	889	2.9	30,234	23,851	78.9
* C. freundii*				28,300	25,808	91.2	28,300	4475	15.8	28,300	2668	9.4	28,300	2012	7.1
* M. morganii*				16,162	15,585	96.4	16,162	5609	34.7	16,162	5799	35.9	16,162	14,296	88.5
* P. stuartii*				7916	7675	97.0	7916	2272	28.7	7916	5957	75.3	7916	6984	88.2
*S. marcsescens*				11,449	10,426	91.1	11,449	337	2.9	11,449	972	8.5	11,449	10,599	92.6

*ESBL* extended-spectrum beta-lactamase-producing phenotype, *NS* not susceptible

Observed rates of resistance varied by characteristics of the clinical setting associated with the ambulatory facility at which the test was performed (urban/rural location, bed size, and teaching status) and by census region (Additional file 1: Table S2). Significant associations between larger hospitals (based on bed size) and higher resistance rates were observed for several pathogen/resistance profiles, including ESBL-producing *E. coli* and *Klebsiella* isolates, beta-lactam-resistant Enterobacterales isolates, TMP/SMX- and FQ-resistant *Klebsiella*, and NTF-resistant non-*E. coli*/*Klebsiella* Enterobacterales isolates). The highest rates of Enterobacterales isolates with an ESBL-producing phenotype were observed in the Pacific and West South Central region.

Resistance to two or more antibiotic classes occurred in 16.4% of isolates and 5.5% were resistant to three or more classes. Of 145,448 isolates with an ESBL-producing phenotype, 70.1%, 59.9%, and 33.5% were NS to FQ, TMP/SMX, and NFT, respectively (Table 2). A total of 30,698 isolates (21.2% of ESBL-producing phenotype isolates and 1.4% of all isolates) were NS to TMP/SMX, FQ, and NFT.

**Table 2:** Multiple antibiotic resistance in ambulatory-onset urine Enterobacterales isolates (2011–2020)

ESBL resistance profile	n	Resistance to additional antimicrobials n (%)			
		FQ	TMP/SMX	NFT	Beta-lactams
ESBL only	145,448 (100%)	101,971 (70.1%)	87,145 (59.9%)	48,470 (33.5%)	134,038 (92.2%)
ESBL + FQ	101,971 (100%)		73,363 (71.9%)	38,372 (37.6%)	101,222 (99.3%)
ESBL + TMP/SMX	87,145 (100%)	73,363 (84.2%)		35,619 (40.9%)	86,738 (99.5%)
ESBL + NFT	48,740 (100%)	38,372 (78.7%)	35,619 (73.1%)		48,497 (99.5%)
ESBL + FQ + TMP/SMX	73,363 (100%)			30,784 (42.0%)	73,030 (99.5%)
ESBL + FQ + TMP/SMX + NFT	30,784 (100%)				30,698 (99.7%)

Percentages are based on the number of isolates with the specified ESBL resistance profile (column labeled “n”)

*ESBL* extended-spectrum beta-lactamase-producing phenotype, *FQ* fluoroquinolones, *NFT* nitrofurantoin, *NS* not susceptible, *TMP/SMX* trimethoprim/sulfamethoxazole

### Trends in antibiotic-resistant urinary Enterobacterales over time

In multivariable adjusted analyses, the percent of isolates with an ESBL-producing phenotype increased by 30% between 2011 and 2020 from 6.5% (95% confidence interval [CI] 6.3–6.8) in 2011 to 9.4% (95% CI 9.1–9.6%) in 2020 (*P* < 0.001) (Table 3). NFT resistance rates showed a curvilinear trend, but the overall increase over time was confirmed in adjusted GEE models. Resistance to NFT increased from 21.6% (95% CI 21.1–22.4) in 2011 to 22.3% (95% CI 21.7–23.1) in 2020 (*P* < 0.001). In contrast, the percent of resistant isolates in the other three antimicrobial resistance groups decreased modestly, but significantly, during this time period (all *P* < 0.001). Resistance to two or more drug classes (from 19.7% [95% CI 19.1–20.0] in 2011 to 18.0% [95% CI, 17.4–18.4] in 2020) and to three or more drug classes (from 6.7% [95% CI 6.5–6.9] in 2011 to 6.5% [95% CI 6.3–6.7] in 2020) also decreased significantly (both *P* < 0.001). We observed significant changes in seasonal patterns that varied by resistance group (Table 3). The highest rates for isolates resistant to beta-lactams, FQ, and TMP-SMX occurred during Q1, whereas the highest ESBL-producing phenotype and NFT NS rates were observed in Q4. Resistance estimates based on characteristics of the clinical facility associated with the site at which the urinary culture was tested are presented in Additional file 1: Table S3.

**Table 3:** All Enterobacterales isolates: adjusted estimates for resistance over time and by geographic region

Characteristics	ESBL		Beta-lactam		Trimethoprim/sulfamethoxazole		Fluoroquinolone		Nitrofurantoin	
	Est (95% CI)	*P*	Est (95% CI)	*P*	Est (95% CI)	*P*	Est (95% CI)	*P*	Est (95% CI)	*P*
Year		< 0.001		< 0.001		< 0.001		< 0.001		< 0.001
2011	6.5 (6.3–6.8)		60.5 (60.2–60.9)		25.7 (25.1–26.2)		25.6 (25.0–26.2)		21.6 (21.1–22.4)	
2012	6.8 (6.5–7.0)		59.9 (59.5–60.3)		26.5 (25.9–27.0)		25.0 (24.4–25.5)		23.1 (22.5–23.9)	
2013	6.9 (6.7–7.1)		59.0 (58.6–59.4)		26.2 (25.6–26.8)		24.2 (23.7–24.8)		22.4 (21.9–23.2)	
2014	7.3 (7.1–7.6)		58.4 (58.0–58.8)		25.7 (25.1–26.2)		23.8 (23.3–24.4)		22.5 (21.9–23.3)	
2015	7.5 (7.3–7.8)		57.7 (57.3–58.1)		25.6 (25.0–26.2)		23.2 (22.7–23.8)		20.9 (20.4–21.8)	
2016	7.8 (7.5–8.0)		57.2 (56.7–57.5)		24.8 (24.2–25.4)		22.7 (22.1–23.3)		18.9 (18.4–19.8)	
2017	8.2 (7.9–8.5)		56.9 (56.5–57.4)		24.6 (23.9–25.2)		22.3 (21.7–23.0)		19.0 (18.4–19.9)	
2018	8.6 (8.3–8.8)		56.6 (56.2–57.0)		24.5 (23.9–25.1)		22.1 (21.5–22.8)		20.2 (19.7–21.1)	
2019	8.9 (8.7–9.2)		56.3 (55.8–56.7)		24.6 (23.9–25.1)		22.2 (21.5–22.8)		21.9 (21.4–22.7)	
2020	9.4 (9.1–9.6)		56.1 (55.6–56.4)		23.9 (23.2–24.4)		21.9 (21.3–22.5)		22.3 (21.7–23.1)	
Season (quarter)		0.015		0.009		< 0.001		< 0.0001		< 0.001
1	8.2 (6.5–9.9)		57.8 (56.7–58.9)		25.4 (24.2–26.2)		23.5 (22.2–24.7)		20.8 (18.6–22.8)	
2	8.1 (6.3–9.9)		57.5 (56.3–58.5)		25.3 (24.1–26.2)		23.1 (21.8–24.3)		20.7 (18.4–22.7)	
3	7.9 (6.2–9.8)		56.9 (55.7–57.9)		24.3 (23.1–25.2)		22.3 (21.0–23.6)		20.9 (18.5–22.9)	
4	8.3 (6.6–10.0)		57.2 (56.1–58.2)		24.7 (23.5–25.6)		22.7 (21.5–23.9)		21.9 (19.7–23.9)	
Census region^a^		0.003		< 0.001		< 0.001		< 0.001		0.005
East North Central	5.7 (3.7–7.6)		55.9 (54.4–57.3)		21.6 (19.8–22.9)		18.7 (16.9–20.5)		20.2 (17.6–23.0)	
East South Central	9.2 (7.2–11.1)		64.2 (62.6–65.7)		29.1 (27.3–30.4)		28.2 (26.3–30.0)		23.1 (20.4–25.7)	
Middle Atlantic	8.1 (6.2–10.1)		58.6 (57.2–60.1)		22.9 (21.1–24.1)		22.4 (20.6–24.2)		21.2 (18.6–24.1)	
Mountain	4.9 (3.0–6.9)		57.3 (55.9–58.8)		22.5 (20.6–23.7)		17.6 (15.8–19.4)		16.6 (14.0–19.2)	
New England	12.1 (10.9–13.3)		65.5 (64.5–66.5)		23.5 (21.9–24.7)		19.6 (18.4–20.9)		18.8 (16.6–21.4)	
Pacific	10.6 (8.6–12.5)		56.1 (54.6–57.6)		25.8 (24.0–27.1)		22.6 (20.8–24.4)		17.9 (15.2–20.5)	
South Atlantic	7.4 (5.4–9.3)		52.5 (51.0–54.0)		23.7 (21.9–25.0)		22.7 (20.9–24.5)		22.9 (20.3–25.6)	
West North Central	8.8 (7.6–10.0)		52.4 (51.4–53.3)		24.3 (22.8–25.5)		24.6 (23.4–25.8)		22.0 (19.9–24.6)	
West South Central	8.6 (6.6–10.5)		54.1 (52.7–55.6)		26.3 (24.4–27.7)		22.7 (20.9–24.5)		21.4 (18.7–24.1)	

*CI* confidence interval, *ESBL* extended-spectrum beta-lactamase-producing phenotype, *Est* estimated, *NS* not susceptible

^a^States included in the data sample by census regions were: East North Central: Illinois, Indiana, Michigan, Ohio, and Wisconsin; East South Central: Alabama, Kentucky, Mississippi, and Tennessee; Middle Atlantic: New Jersey, New York, and Pennsylvania; Mountain: Arizona, Idaho, Montana, and New Mexico; New England: Connecticut and New Hampshire; Pacific: California, Oregon, and Washington; South Atlantic: Delaware, Georgia, Florida, Maryland, North Carolina, South Carolina, Washington D.C., West Virginia, and Virginia; West North Central: Iowa and Missouri; West South Central: Louisiana, Oklahoma, Texas

### Trends in antibiotic-resistant urinary Enterobacterales by pathogen

Multivariable adjusted subgroup analyses by pathogen found that resistance trends, seasonal patterns, and geographic variations for *E. coli* were generally similar to those observed for all resistant urinary Enterobacterales isolates (Table 4). Specifically, an increasing trend over the years was observed for ESBL-producing phenotype (*P* < 0.001) and decreasing trends were observed for beta-lactam, TMP/SMX, and FQ resistance (all *P* < 0.03). In contrast to the increased resistance to NFT observed in all Enterobacterales urinary isolates, *E. coli* isolates showed reduced resistance to NFT over time (*P* < 0.001). In addition, although the proportion of *E. coli* isolates resistant to two or more drug classes showed a slight but significant decrease during this time period (from 17.8% [95% CI 17.3–18.1] in 2011 to 17.6 [95% CI 17.2–17.9] in 2020; *P* = 0.027), a significant increase was observed in the proportion of *E. coli* isolates resistant to three or more drug classes (from 4.3% [95% CI 4.2–4.4] in 2011 to 5.6% [95% CI 5.5–5.7] in 2020; *P* < 0.001).

**Table 4:** *E. coli* isolates: adjusted estimates for resistance over time and by geographic region

Characteristics	ESBL		Beta-lactam		Trimethoprim/sulfamethoxazole		Fluoroquinolones		Nitrofurantoin	
	Est (95% CI)	*P*	Est (95% CI)	*P*	Est (95% CI)	*P*	Est (95% CI)	*P*	Est (95% CI)	*P*
Year		< 0.001		< 0.001		0.029		< 0.001		< 0.001
2011	6.3 (6.0–6.6)		54.7 (53.9–55.5)		27.7 (27.2–28.2)		26.0 (25.3–26.6)		4.5 (3.4–5.5)	
2012	6.6 (6.3–6.9)		54.1 (53.3–54.9)		29.3 (28.8–29.8)		27.0 (26.4–27.5)		6.5 (5.4–7.5)	
2013	6.8 (6.6–7.1)		53.4 (52.6–54.1)		28.9 (28.4–29.4)		26.5 (25.9–27.0)		6.2 (5.1–7.1)	
2014	7.4 (7.1–7.7)		52.9 (52.1–53.7)		28.8 (28.3–29.3)		26.3 (25.7–26.8)		6.2 (4.9–7.2)	
2015	7.7 (7.4–8.0)		52.1 (51.4–52.9)		28.5 (27.9–29.0)		25.8 (25.2–26.3)		4.6 (2.9–5.7)	
2016	8.1 (7.8–8.4)		51.5 (50.6–52.3)		27.6 (27.1–28.1)		25.5 (24.9–26.0)		3.5 (1.9–4.6)	
2017	8.6 (8.3–8.9)		51.2 (50.4–52.1)		27.7 (27.1–28.2)		25.3 (24.7–25.9)		3.5 (1.9–4.6)	
2018	9.1 (8.8–9.4)		50.9 (50.1–51.8)		28.1 (27.6–28.7)		25.3 (24.7–26.0)		3.9 (2.3–5.0)	
2019	9.7 (9.4–10.0)		50.8 (49.9–51.5)		28.6 (28.0–29.2)		25.6 (24.9–26.2)		4.3 (2.7–5.4)	
2020	9.8 (9.5–10.2)		50.6 (49.7–51.4)		27.3 (26.7–27.8)		25.7 (25.0–26.3)		4.1 (2.4–5.2)	
Season (quarter)		0.009		< 0.001		0.001		< 0.001		0.031
1	8.6 (6.3–10.8)		52.6 (51.6–53.6)		28.6 (27.3–29.4)		26.4 (26.2–26.6)		4.6 (3.3–6.4)	
2	8.4 (6.2–10.8)		52.0 (51.0–53.0)		28.5 (27.1–29.3)		26.2 (26.0–26.5)		4.6 (3.3–6.4)	
3	8.3 (6.0–10.7)		51.0 (49.9–52.0)		27.6 (26.2–28.5)		25.1 (24.9–25.4)		4.3 (3.0–6.1)	
4	8.7 (6.5–11.0)		51.5 (50.5–52.5)		27.9 (26.5–28.6)		25.5 (25.2–25.7)		4.4 (3.0–6.2)	
Census region		< 0.001		< 0.001		< 0.001		< 0.001		0.044
East North Central	5.7 (3.1–8.2)		47.4 (45.6–49.2)		24.5 (22.5–25.6)		20.8 (19.9–21.6)		3.9 (2.6–5.9)	
East South Central	9.8 (7.2–12.3)		59.1 (57.2–60.9)		33.1 (31.2–34.3)		32.2 (31.3–33.0)		5.4 (3.9–7.2)	
Middle Atlantic	8.4 (5.8–10.9)		51.1 (49.4–52.9)		25.4 (23.5–26.5)		24.1 (23.2–24.9)		4.5 (3.1–6.4)	
Mountain	5.1 (2.6–7.6)		48.4 (46.7–50.2)		23.7 (21.9–24.9)		20.3 (19.4–21.1)		3.8 (2.4–5.6)	
New England	11.8 (10.3–13.4)		61.5 (60.1–62.8)		26.4 (25.1–27.7)		21.3 (20.5–22.0)		2.7 (2.1–3.5)	
Pacific	10.8 (8.3–13.4)		49.8 (48.0–51.5)		27.7 (25.8–28.9)		24.5 (23.6–25.2)		4.2 (2.8–6.2)	
South Atlantic	7.6 (5.1–10.2)		47.4 (45.7–49.2)		27.7 (25.8–28.8)		26.6 (25.7–27.4)		4.6 (3.3–6.5)	
West North Central	11.6 (10.1–13.2)		46.1 (44.8–47.4)		26.2 (24.8–27.5)		26.6 (25.7–27.3)		2.8 (2.1–4.8)	
West South Central	9.2 (6.6–11.7)		53.2 (51.4–55.0)		30.6 (28.7–31.8)		26.5 (25.6–27.3)		4.4 (3.0–6.4)	

*CI* confidence interval, *ESBL* extended-spectrum beta-lactamase-producing phenotype, *Est* estimated, *NS* not susceptible

*Klebsiella* isolates (*K. pneumoniae*, *K. oxytoca*, and *K. aerogenes*) accounted for 16.3% (363,029/2,228,515) of isolates analyzed for beta-lactam, FQ, NFT, and TMP/SMX resistance and 15.9% (332,795/2,095,447) of isolates in ESBL-producing phenotype analyses, which did not include *K. aerogenes* (Table 1). With the exception of beta-lactams, the percent of *Klebsiella* isolates with resistant phenotypes increased over time for all drug classes (all *P* < 0.001) (Table 5). Increases were also observed in the proportion of isolates resistant to three or more drugs, but not for two or more drugs (data not shown). Significant changes in seasonal patterns were found for beta-lactam- and NFT-NS *Klebsiella* isolates (*P* = 0.04 and < 0.001, respectively), but not for ESBL-producing phenotype or TMP/SMX- or FQ-NS isolates. Compared with 2020 resistance rates observed in the analysis of all Enterobacterales isolates (Table 3), *Klebsiella* showed substantially higher rates of resistance to beta-lactams (81.1% [95% CI 80.5–81.7] vs 56.1% [95% CI 55.6–56.4) and NFT (59.2% [95% CI 57.9–60.6] vs 22.3% [95% CI 21.7–23.1]), but lower rates of resistance to FQ (6.8% [95% CI 5.6–7.1] vs 21.9% [95% CI 21.3–22.5]) and TMP/SMX (11.3% [95% CI 11.1–11.5] vs 23.9% [95% CI 23.2–24.4]) and lower rates of ESBL-producing phenotype (7.2% [95% CI 7.0–7.3] vs 9.4% [95% CI 9.1–9.6]).

**Table 5:** *Klebsiella* spp. isolates^a^: adjusted estimates for resistance over time and by geographic region

Characteristics	ESBL		Beta-lactam		Trimethoprim–sulfamethoxazole		Fluoroquinolones		Nitrofurantoin	
	Est (95% CI)	*P*	Est (95% CI)	*P*	Est (95% CI)	*P*	Est (95% CI)	*P*	Est (95% CI)	*P*
Year		< 0.001		< 0.001		< 0.001		< 0.001		< 0.001
2011	6.3 (6.2–6.4)		92.8 (92.3–93.4)		11.0 (10.9–11.2)		6.7 (4.6–8.0)		58.5 (57.1–59.7)	
2012	6.4 (6.2–6.5)		91.4 (90.9–91.9)		10.4 (10.2–10.6)		6.9 (4.9–8.2)		60.0 (58.6–61.2)	
2013	6.4 (6.3–6.5)		89.5 (88.9–90.0)		9.9 (9.6–10.1)		6.9 (4.9–8.2)		60.8 (59.4–62.0)	
2014	6.6 (6.4–6.7)		87.7 (87.1–88.2)		9.3 (9.0–9.5)		5.6 (3.4–6.9)		59.8 (58.4–61.0)	
2015	6.6 (6.5–6.7)		86.8 (86.2–87.3)		10.5 (10.2–10.7)		6.2 (5.0–7.5)		57.2 (55.8–58.4)	
2016	6.6 (6.5–6.7)		85.8 (85.3–86.4)		10.4 (10.2–10.6)		5.9 (4.7–7.1)		52.5 (51.2–53.9)	
2017	6.7 (6.6–6.9)		84.9 (84.3–85.5)		10.3 (10.0–10.5)		5.9 (4.7–7.1)		53.4 (52.0–54.8)	
2018	6.8 (6.7–7.0)		84.0 (83.5–84.6)		10.6 (10.3–10.8)		6.0 (4.8–7.2)		54.7 (53.3–56.1)	
2019	6.9 (6.8–7.1)		82.8 (82.2–83.4)		11.4 (11.1–11.6)		6.3 (5.1–7.6)		55.4 (54.0–56.8)	
2020	7.2 (7.0–7.3)		81.1 (80.5–81.7)		11.3 (11.1–11.5)		6.8 (5.6–7.1)		59.2 (57.9–60.6)	
Season (quarter)		0.336		0.038		0.310		0.060		< 0.001
1	6.9 (6.1–7.8)		86.1 (81.9–90.3)		10.8 (9.2–12.1)		6.5 (5.7–7.4)		55.8 (51.3–60.1)	
2	6.8 (6.0–7.7)		85.6 (81.1–89.8)		10.5 (9.0–11.9)		6.3 (5.4–7.1)		55.4 (50.6–59.7)	
3	6.7 (5.9–7.5)		85.1 (80.9–89.3)		10.4 (8.8–11.8)		6.1 (5.2–6.9)		57.2 (52.3–61.5)	
4	6.6 (5.8–7.4)		84.8 (80.3–89.0)		10.7 (9.1–12.2)		6.2 (5.3–7.1)		57.7 (53.0–62.0)	
Census region		0.003		< 0.001		0.050		< 0.001		0.033
East North Central	5.1 (4.1–6.0)		93.5 (88.8–98.2)		9.2 (7.3–10.6)		6.2 (5.2–7.2)		54.2 (48.5–59.6)	
East South Central	7.2 (6.2–8.3)		91.4 (86.3–96.2)		11.6 (10.0–13.0)		6.8 (5.7–7.8)		58.0 (52.3–62.7)	
Middle Atlantic	7.1 (6.1–8.1)		93.3 (88.6–98.0)		10.7 (9.0–12.2)		5.4 (4.3–6.4)		56.6 (50.9–61.9)	
Mountain	4.7 (3.8–5.7)		86.4 (81.7–91.2)		8.1 (6.6–9.5)		3.6 (2.9–4.6)		52.6 (46.9–57.8)	
New England	3.6 (3.0–4.2)		85.2 (82.3–88.1)		7.9 (7.1–8.8)		7.0 (5.9–8.1)		56.1 (52.0–61.3)	
Pacific	8.6 (7.6–9.6)		87.6 (82.9–92.3)		10.7 (8.8–12.1)		7.0 (5.9–8.0)		56.4 (50.7–61.8)	
South Atlantic	7.0 (6.0–7.9)		74.7 (70.0–79.5)		10.7 (8.9–12.0)		7.0 (5.9–8.0)		61.6 (56.0–67.1)	
West North Central	3.6 (2.9–4.3)		94.9 (91.9–97.7)		7.8 (6.3–9.0)		2.0 (1.1–3.0)		48.2 (43.9–53.4)	
West South Central	7.2 (6.2–8.1)		69.4 (64.6–74.1)		11.7 (9.7–13.3)		6.4 (5.3–7.5)		55.6 (50.0–61.1)	

*CI* confidence interval, *ESBL* extended-spectrum beta-lactamase-producing phenotype, *Est* estimated, *NS* not susceptible

^a^*K. pneumoniae*, *K. oxytoca*, and *K. aerogenes*

“Other” (non-*E. coli*, non-*Klebsiella*) Enterobacterales isolates included *C. freundii, E. cloacae, M. morganii, P. mirabilis, P. stuartii,* and *S. marcescens* and accounted for 242,038 (10.9%) of all isolates (Table 1, Additional file 1: Table S4). This subgroup of Enterobacterales isolates had different resistance profiles from Enterobacterales isolates as a whole, most notably in NFT NS isolates (75.6% [95% CI 74.4–76.9] in 2020 for “other” isolates compared with 22.3% [95% CI 21.7–23.1] for all Enterobacterales isolates). The proportion of these isolates resistant to two or more drug classes or to three or more drugs classes decreased significantly during this time period (data not shown).

## Discussion

In this study of ambulatory US patients, over half of Enterobacterales-positive urinary cultures were due to organisms resistant to at least one antibiotic class. Although it was encouraging to note a downward trend in resistance to beta-lactams, TMP/SMX, and FQ among Enterobacterales isolates over time, rates were only lower by 2–5% over the 10-year period and suggest that resistance to these classes is firmly established in the community. More disconcerting is the increase observed in NFT NS isolates and particularly in ESBL-producing phenotype isolates, which increased by approximately 30% during the 10-year span. FQ resistance rates (21.9% [95% CI 21.3–22.5] in 2020) remained well over the 10% threshold recommended by IDSA guidelines as the cut-off for empiric therapy for acute pyelonephritis and TMP/SMX resistance rates (23.9% [95% CI 23.2–24.4] in 2020) similarly exceeded the recommended 20% resistance threshold for use of this agent in acute cystitis [3]. The current IDSA guidelines are from 2010 and do not provide resistance thresholds for other drugs that are now commonly used to treat UTIs. However, if the highest IDSA threshold of 20% is applied to the other antibiotic classes in our analysis, then all four of the drug classes evaluated (beta-lactam, FQ, TMP/SMX, and NTF) currently have national resistance rates too high for empiric use in the management of UTIs. Also noteworthy were the very high rates of resistance of non-*E. coli* urinary Enterobacterales isolates, which accounted for 27.2% of isolates, to recommended first-line agents, including NFT and beta-lactams, and increases in resistance to three or more antibiotic classes in *E. coli* and *Klebsiella* spp. These resistance trends have the potential to impair effective empiric management of outpatient UTIs and negatively impact patient outcomes. Our findings support the importance of careful review of urine culture and susceptibility results with modification of empiric treatment as needed. Highlighting this point, a recent single-center study found that outpatient empiric UTI therapy required modification in 26% of patients, primarily due to antimicrobial resistance to the initial agent [17].

Our data are consistent with increases in Enterobacterales isolates with ESBL-producing phenotypes noted in a regional study of outpatients in the southeastern US [18] and in hospitalized US patients [15, 19], as well as in *E. coli* urinary isolates in Canadian [6] and US outpatients [7]. Together, these findings indicate that Enterobacterales isolates with an ESBL-producing phenotype remain an important concern for patients with community-acquired UTIs. Although some risk factors for UTIs due to ESBL-producing pathogens have been proposed, including recent hospital stay or antibiotic treatment [20–22], patients with community-acquired UTIs due to an ESBL-producing organism may present with no identifiable risk factors [8].

Outpatient UTIs caused by Enterobacterales isolates with an ESBL-producing phenotype are associated with a sevenfold increase in clinical failure compared with non-ESBL-producing isolates [9], and inpatients with UTIs due to ESBL-producing isolates have a longer hospital length of stay, higher mortality, and higher rates of re-admission [22, 23]. Due to the morbidity and mortality associated with UTIs due to ESBL-producing organisms, prompt, effective treatment is essential to improving patient outcomes. However, discordant empiric therapy is common in both outpatients and inpatients with UTIs caused by isolates with an ESBL-producing phenotype [8, 9, 23]. As shown in our analyses, Enterobacterales isolates with an ESBL-producing phenotype are often highly resistant to oral agents commonly used to treat UTIs. Although the isolates generally retain susceptibility to carbapenems [24], these agents are currently only available in intravenous formulations and hospitalization is often required for their use. Additional oral options, including oral penems, would provide valuable alternatives to currently available antibiotics used to treat outpatient UTIs.

Although our study did not explore the association between antibiotic use and antibiotic-resistant UTIs, a large body of evidence suggests that community antibiotic use has a strong influence on antibiotic resistance rates [25, 26]. It is therefore possible that decreases in beta-lactams, FQ, and TMP-SMX over time reflect more judicious use of these drugs in the ambulatory setting.

Our study encompasses data from 2020, a time during which the coronavirus disease-2019 (COVID-19) pandemic altered outpatient management [27]. Many healthcare systems view telemedicine as an answer to resource optimization, particularly during surge capacity times such as cold/flu season and more recently the COVID-19 pandemic. However, recent data have shown that while virtual visits may decrease operational costs, the prescribing of antimicrobials often increases with telemedicine [28]. The acquisition of urine cultures also appears to be less frequent for virtual visits [2]. It is therefore possible that the virtual medicine trend augmented by COVID-19 restrictions may impact uUTI management, including potential increases in use of inappropriate antimicrobials and reductions in performing urine cultures. Although we did not observe increased resistance in 2020 urinary Enterobacterales isolates, we did document a reduction in the number of ambulatory cultures analyzed in 2020 compared with 2018–2019, despite the fact that the number of study sites increased. Future analyses will be required to address the underlying reasons for this observation as well as potential effects on patient outcomes.

Seasonal variations in Enterobacterales antimicrobial resistance were identified. Higher rates of resistance lag approximately 1 month behind increased antibiotic usage [29], and it is therefore likely that this seasonality relates to the increased use of oral antibiotics during the influenza season, as has been suggested in other studies [30–32]; this observation warrants further evaluation.

Although we conducted explorative analyses on the association between hospital characteristics of associated testing facilities and antimicrobial resistance, it should be emphasized that the patients in this study were not hospitalized. Accordingly, the data on geographic variations have the most relevance to the population being examined. In addition to geographic variations, significantly higher resistance rates were observed for testing facilities associated with larger hospitals (> 300 beds) for some pathogens/resistance profiles, including ESBL-producing phenotypes for *E. coli* and *Klebsiella* isolates. This observation may relate to a higher likelihood of antimicrobial resistance in more urban areas, but more study will be needed to confirm this connection. Associations with other hospital characteristics were inconsistent and so their potential clinical significance is unclear.

Conservation of effective antimicrobials is an increasingly supported tenet of antimicrobial stewardship, which has recently gained traction in the US with inclusion as an inpatient Centers for Medicare & Medicaid Services Condition of Participation metric and new outpatient standards from The Joint Commission [33]. Through stewardship programs, clinicians are now expected to weigh the risk/benefit ratio of empiric therapy on an individual level while taking into account the aggregate effect on population resistance. In some cases, they are assisted in that decision by the use of electronic medical record-based clinical decision tools designed to aid clinician prescribing for common clinical syndromes such as uUTI and respiratory infections [34, 35]. Nevertheless, the balance between appropriate guideline-endorsed empiric therapy for individual patients and potential aggregate public health issues related to bulk prescribing remains a complicated issue. More complete information on outpatient resistance trends may help the clinician when choosing an empiric uUTI agent. This, coupled with efforts to improve both the development and availability of better diagnostic tests, may in turn enable clinicians to treat based on the individual while taking into account the larger public health consequences of antimicrobial resistance.

Limitations of our study include the identification of non-duplicate culture-positive isolates rather than confirmed clinical infections. Although classification of urinary pathogens versus commensal bacteria is an ongoing area of research [36], the bacteria evaluated in this study are all known to have pathogenic potential and are typically considered pathogens when found in urine. Similarly, our data did not capture whether the affected patient had cystitis or pyelonephritis. The study was not designed to evaluate clinical outcomes associated with positive urinary cultures. Antimicrobial susceptibility results relied on local microbiology practices at each facility and were not standardized across facilities. Enterobacterales testing practices and antibiotic breakpoints are known to vary among different institutions, including susceptibility criteria for ESBL [37], FQ [38], and beta-lactams [39]. Finally, ordering a urine culture is not a common practice for uUTI and in particular in patients who do not have recurring UTIs; the data therefore may more heavily represent a certain subset of the UTI population. Nevertheless, the resistance trends reported here highlight the need for augmented surveillance of local susceptibility patterns to better inform empiric therapy options.

## Conclusions

Our data provide contemporary insights into antimicrobial resistance trends in the US and document high rates of antimicrobial resistance and increasing ESBL positivity rates in Enterobacterales isolates in urinary cultures from ambulatory patients. These data on antimicrobial resistance may be of value when considering empiric therapy options for patients with UTIs and will serve as valuable benchmarks for antimicrobial stewardship efforts in the outpatient setting.

## Supplementary Information

**Additional file 1: Table S1.** Distribution of study sites. **Table S2.** Summary statistics (unadjusted) of resistance in ambulatory-onset urinary Enterobacterales isolates over time and by hospital characteristics of the facility associated with the outpatient setting at which the urine culture was collected. % NS data presented as quarterly mean (SD). **Table S3.** Adjusted estimates for percent of resistance by hospital characteristics and by pathogen. **Table S4.** Other Enterobacterales organisms excluding *E. coli* and *Klebsiella* spp: adjusted estimates for resistance over time and by geographic region.

## Data Availability

The datasets used and/or analyzed during the current study are included in the tables in the main manuscript and additional information.

## Abbreviations

COVID-19: Coronavirus disease-2019
ESBL: Extended-spectrum beta-lactamase
ESC: Extended-spectrum cephalosporins
FDA: Food and Drug Administration
FQ: Fluoroquinolones
I: Intermediate
IDSA: Infectious Diseases Society of America
NS: Not susceptible
NFT: Nitrofurantoin
Q: Quarter
R: Resistant
TMP/SMZ: Trimethoprim/sulfamethoxazole
UTI: Urinary tract infection
uUTI: Uncomplicated urinary tract infection
