# Supplementary material for: A multicenter analysis of trends in resistance in urinary Enterobacterales isolates from ambulatory patients in the United States: 2011–2020
Source: BMC Infect Dis. 2022 Feb 28;22:194. doi: 10.1186/s12879-022-07167-y (PMC8883240; doi:10.1186/s12879-022-07167-y)
Supplement: Supplementary file 1 — Additional file 1: Table S1. Distribution of study sites. Table S2. Summary statistics (unadjusted) of resistance in ambulatory-onset urinary Enterobacterales isolates over time and by hospital characteristics of the facility associated with the outpatient setting at which the urine culture was collected. % NS data presented as quarterly mean (SD). Table S3. Adjusted estimates for percent of resistance by hospital characteristics and by pathogen. Table S4. Other Enterobacterales organisms excluding E. coli and Klebsiella spp: adjusted estimates for resistance over time and by geographic region. [file 12879_2022_7167_MOESM1_ESM.docx]

**Supplementary Information**

**A multicenter analysis of trends in resistance in urinary Enterobacterales isolates from ambulatory patients in the United States: 2011 – 2020**

Michael W. Dunne, Steven I. Aronin, Kalvin C. Yu, Janet A. Watts, Vikas Gupta

**Supplementary Table 1** Distribution of study sites. Bed size, teaching status, and facility type refer to characteristics of the hospitals affiliated with the outpatient center at which the urine culture was collected.

| **Characteristics** | **Number of sites** | |
| --- | --- | --- |
|  | **n** | **%** |
| **Overall** | 338 | 100.0 |
| **Year**^a^ |  |  |
| 2011 | 97 | 28.7 |
| 2012 | 115 | 34.0 |
| 2013 | 136 | 40.2 |
| 2014 | 162 | 47.9 |
| 2015 | 192 | 56.8 |
| 2016 | 227 | 67.2 |
| 2017 | 249 | 73.7 |
| 2018 | 287 | 84.9 |
| 2019 | 320 | 94.7 |
| 2020 | 338 | 100.0 |
| **Bed size** |  |  |
| 0-100 | 116 | 34.3 |
| 100-300 | 138 | 40.8 |
| 300 over | 84 | 24.9 |
| **Urban/Rural** |  |  |
| Rural | 116 | 34.3 |
| Urban | 222 | 65.7 |
| **Teaching status** |  |  |
| Non-teaching | 229 | 67.8 |
| Teaching | 109 | 32.2 |
| **Facility type** |  |  |
| Short Term | 297 | 87.9 |
| Critical | 22 | 6.5 |
| Long Term | 6 | 1.8 |
| Rehabilitation | 4 | 1.2 |
| Psychiatric | 2 | 0.6 |
| **Census region**^b^ |  |  |
| East North Central | 47 | 13.9 |
| East South Central | 50 | 14.8 |
| Middle Atlantic | 57 | 16.9 |
| Mountain | 13 | 3.8 |
| New England | 6 | 1.8 |
| Pacific | 34 | 10.1 |
| South Atlantic | 51 | 15.1 |
| West North Central | 16 | 4.7 |
| West South Central | 64 | 18.9 |

^a^ The numbers of sites across years do not add up to 338 and may change from year to year.

^b^ States included in the data sample by census regions were:

East North Central: Illinois, Indiana, Michigan, Ohio, and Wisconsin

East South Central: Alabama, Kentucky, Mississippi, and Tennessee

Middle Atlantic: New Jersey, New York, and Pennsylvania

Mountain: Arizona, Idaho, Montana, and New Mexico

New England: Connecticut and New Hampshire

Pacific: California, Oregon, and Washington

South Atlantic: Delaware, Georgia, Florida, Maryland, North Carolina, South Carolina, Washington D.C., West Virginia, and Virginia

West North Central: Iowa and Missouri

West South Central: Louisiana, Oklahoma, Texas

**Supplementary Table 2** Summary statistics (unadjusted) of resistance in ambulatory-onset urinary Enterobacterales isolates over time and by hospital characteristics of the facility associated with the outpatient setting at which the urine culture was collected. % NS data presented as quarterly mean (SD).

| **Charac-teristics** | **Antimicrobial resistance** | | | | | | | | | | | | | | |
| --- | --- | --- | --- | --- | --- | --- | --- | --- | --- | --- | --- | --- | --- | --- | --- |
|  | **ESBL** | | | **Beta-lactam** | | | **Trimethoprim/ sulfamethoxazole** | | | **Fluoroquinolones** | | | **Nitrofurantoin** | | |
|  | **Tested** | **NS** | **% NS** | **Tested** | **NS** | **% NS** | **Tested** | **NS** | **% NS** | **Tested** | **NS** | **%NS** | **Tested** | **NS** | **% NS** |
| **All** | 2,095,447 | 145,448 | 7.6 (7.4) | 2,228,515 | 1,280,780 | 58.3 (12.3) | 2,228,515 | 513,945 | 24.7 (8.7) | 2,228,515 | 459,339 | 22.1 (10.0) | 2,228,515 | 450,818 | 21.3 (9.3) |
| **Year** |  |  |  |  |  |  |  |  |  |  |  |  |  |  |  |
| 2011 | 80,628 | 3,723 | 5.1 (5.0) | 85,775 | 50,686 | 59.9 (10.1) | 85,775 | 20,577 | 25.2 (7.7) | 85,775 | 18,993 | 23.2 (9.4) | 85,775 | 17,816 | 22.1 (9.4) |
| 2012 | 93,050 | 4,637 | 5.6 (5.6) | 98,993 | 58,717 | 59.5 (10.5) | 98,993 | 23,769 | 26.0 (8.5) | 98,993 | 22,250 | 24.1 (10.5) | 98,993 | 21,732 | 23.5 (9.9) |
| 2013 | 115,097 | 6,096 | 6.0 (5.9) | 122,422 | 73,410 | 60.6 (10.9) | 122,422 | 29,788 | 26.1 (8.9) | 122,422 | 27,176 | 24.0 (10.8) | 122,422 | 27,025 | 23.0 (10.4) |
| 2014 | 145,582 | 9,533 | 6.9 (8.8) | 154,198 | 90,927 | 60.3 (11.7) | 154,198 | 37,645 | 25.6 (7.6) | 154,198 | 34,321 | 23.3 (9.3) | 154,198 | 33,967 | 23.2 (10.9) |
| 2015 | 194,820 | 13,007 | 7.4 (9.6) | 206,759 | 120,243 | 58.9 (12.0) | 206,759 | 49,284 | 25.5 (9.1) | 206,759 | 44,820 | 23.3 (9.9) | 206,759 | 41,687 | 21.3 (8.6) |
| 2016 | 252,162 | 17,081 | 7.6 (9.3) | 268,064 | 154,703 | 58.8 (10.7) | 268,064 | 62,246 | 24.5 (7.9) | 268,064 | 57,301 | 22.5 (8.9) | 268,064 | 50,689 | 19.1 (7.2) |
| 2017 | 293,817 | 20,636 | 7.7 (8.6) | 311,950 | 177,101 | 58.0 (11.4) | 311,950 | 70,682 | 24.3 (8.5) | 311,950 | 62,164 | 21.5 (9.5) | 311,950 | 57,785 | 18.9 (7.3) |
| 2018 | 308,831 | 22,257 | 7.7 (4.9) | 328,052 | 188,057 | 57.9 (11.3) | 328,052 | 74,521 | 24.2 (8.1) | 328,052 | 63,652 | 20.7 (9.1) | 328,052 | 63,927 | 20.2 (8.0) |
| 2019 | 313,562 | 24,098 | 8.6 (7.1) | 333,935 | 191,025 | 58.2 (12.7) | 333,935 | 75,503 | 24.6 (9.3) | 333,935 | 64,755 | 21.4 (10.5) | 333,935 | 67,462 | 21.8 (10.4) |
| 2020 | 297,898 | 24,380 | 8.8 (6.1) | 318,367 | 175,911 | 55.2 (15.7) | 318,367 | 69,930 | 23.7 (9.9) | 318,367 | 63,907 | 21.2 (10.8) | 318,367 | 68,728 | 22.3 (10.0) |
| **Bed size** |  |  |  |  |  |  |  |  |  |  |  |  |  |  |  |
| <100 | 267,786 | 20,262 | 7.5 (7.5) | 285,672 | 163,082 | 57.9 (14.2) | 285,672 | 70,215 | 24.7 (10.6) | 285,672 | 64,761 | 22.7 (11.5) | 285,672 | 57,818 | 20.7 (10.8) |
| 100-300 | 862,870 | 57,235 | 7.2 (5.8) | 918,751 | 520,230 | 57.8 (11.3) | 918,751 | 209,005 | 24.4 (8.0) | 918,751 | 188,715 | 21.8 (9.8) | 918,751 | 187,373 | 21.7 (8.8) |
| >300 | 964,791 | 67,951 | 8.2 (9.3) | 1,024,092 | 597,468 | 59.3 (11.9) | 1,024,092 | 234,725 | 25.3 (8.0) | 1,024,092 | 205,863 | 22.0 (8.8) | 1,024,092 | 205,627 | 21.2 (8.6) |
| **Urban/**  **Rural** |  |  |  |  |  |  |  |  |  |  |  |  |  |  |  |
| Urban | 711,312 | 45,777 | 7.2 (5.5) | 758,537 | 426,277 | 58.0 (11.5) | 758,537 | 168,819 | 24.3 (8.2) | 758,537 | 151,666 | 22.6 (10.0) | 758,537 | 149,645 | 20.4 (8.3) |
| Rural | 1,384,135 | 99,671 | 7.8 (8.3) | 1,469,978 | 854,503 | 58.4 (12.7) | 1,469,978 | 345,126 | 25.0 (9.0) | 1,469,978 | 307,673 | 21.9 (10.0) | 1,469,978 | 301,173 | 21.7 (9.8) |
| **Teaching status** |  |  |  |  |  |  |  |  |  |  |  |  |  |  |  |
| Non-teaching | 1,235,143 | 85,202 | 7.3 (6.3) | 1,315,660 | 752,534 | 57.5 (12.8) | 1,315,660 | 301,029 | 24.5 (9.1) | 1,315,660 | 277,640 | 22.3 (10.4) | 1,315,660 | 267,585 | 21.1 (9.6) |
| Teaching | 860,304 | 60,246 | 7.9 (9.0) | 912,855 | 528,246 | 59.6 (11.3) | 912,855 | 212,916 | 25.1 (8.0) | 912,855 | 181,699 | 21.7 (9.3) | 912,855 | 183,233 | 21.5 (8.8) |
| **Census region** |  |  |  |  |  |  |  |  |  |  |  |  |  |  |  |
| East North Central | 428,762 | 23,876 | 5.9 (3.9) | 456,535 | 250,740 | 56.0 (10.4) | 456,535 | 93,670 | 22.0 (6.7) | 456,535 | 78,924 | 19.5 (7.5) | 456,535 | 85,829 | 20.6 (8.6) |
| East South Central | 268,695 | 20,979 | 8.1 (5.5) | 287,273 | 183,089 | 64.1 (9.9) | 287,273 | 78,009 | 28.1 (7.1) | 287,273 | 73,070 | 26.7 (10.2) | 287,273 | 64,715 | 22.6 (8.7) |
| Middle Atlantic | 378,482 | 25,279 | 7.8 (7.7) | 402,961 | 231,231 | 58.3 (11.5) | 402,961 | 87,171 | 23.2 (9.5) | 402,961 | 83,603 | 21.8 (11.2) | 402,961 | 78,901 | 21.0 (10.1) |
| Mountain | 75,389 | 4,115 | 5.2 (4.2) | 80,246 | 45,181 | 56.6 (10.1) | 80,246 | 16,787 | 21.3 (7.9) | 80,246 | 14,102 | 17.7 (6.8) | 80,246 | 14,701 | 18.1 (6.3) |
| New England | 39,187 | 1,935 | 6.6 (14.2) | 41,771 | 20,924 | 60.1 (23.3) | 41,771 | 6,933 | 18.5 (12.9) | 41,771 | 5,258 | 14.3 (13.3) | 41,771 | 8,064 | 19.4 (5.4) |
| Pacific | 243,709 | 22,095 | 9.1 (13.1) | 256,867 | 142,918 | 55.9 (11.1) | 256,867 | 61,490 | 24.4 (7.3) | 256,867 | 51,900 | 20.3 (8.1) | 256,867 | 46,869 | 18.6 (6.2) |
| South Atlantic | 299,544 | 20,119 | 6.9 (3.6) | 318,429 | 182,699 | 58.1 (11.8) | 318,429 | 72,401 | 24.4 (5.7) | 318,429 | 68,087 | 21.7 (6.2) | 318,429 | 75,794 | 23.8 (6.8) |
| West North Central | 35,822 | 2,107 | 6.6 (7.7) | 38,286 | 19,761 | 50.6 (15.7) | 38,286 | 7,494 | 20.9 (12.6) | 38,286 | 5,917 | 17.3 (17.9) | 38,286 | 6,960 | 19.4 (13.9) |
| West South Central | 325,857 | 24,943 | 8.4 (8.1) | 346,147 | 204,237 | 56.7 (14.3) | 346,147 | 89,990 | 27.0 (10.8) | 346,147 | 78,478 | 23.2 (10.4) | 346,147 | 68,985 | 21.5 (11.8) |

*ESBL* extended-spectrum beta-lactamase-producing phenotype, *NS* not susceptible, *SD* standard deviation

**Supplementary Table 3** Adjusted estimates for percent of resistance by pathogen and by hospital characteristics of the facility associated with the outpatient setting at which the urine culture was collected

| **Characteristics** | **ESBL** | | **Beta-lactam** | | **Trimethoprim/ sulfamethoxazole** | | **Fluoroquinolone** | | **Nitrofurantoin** | |
| --- | --- | --- | --- | --- | --- | --- | --- | --- | --- | --- |
|  | **Est (95% CI)** | ***P*** | **Est (95% CI)** | ***P*** | **Est (95% CI)** | ***P*** | **Est (95% CI)** | ***P*** | **Est (95% CI)** | ***P*** |
| **All Enterobacterales isolates** |  |  |  |  |  |  |  |  |  |  |
| **Bed size** |  | 0.076 |  | 0.011 |  | 0.866 |  | 0.348 |  | 0.179 |
| 0-100 | 7.9 (5.9-9.8) |  | 56.4 (54.9-57.9) |  | 25.0 (23.2-26.5) |  | 23.0 (21.2-24.8) |  | 20.8 (18.2-23.5) |  |
| 100-300 | 7.7 (5.7-9.6) |  | 57.6 (56.0-59.0) |  | 24.8 (22.9-26.1) |  | 22.5 (20.6-24.3) |  | 20.9 (18.3-23.6) |  |
| 300 over | 9.1 (7.1-11.0) |  | 57.9 (56.5-59.4) |  | 25.1 (23.3-26.4) |  | 23.3 (21.6-25.1) |  | 21.4 (18.7-24.2) |  |
| **Urban/Rural** |  | 0.227 |  | 0.518 |  | 0.618 |  | 0.769 |  | 0.057 |
| Rural | 7.7 (5.8-9.7) |  | 57.3 (55.7-58.7) |  | 24.9 (23.1-26.2) |  | 23.4 (21.5-25.2) |  | 20.2 (17.6-23.0) |  |
| Urban | 8.4 (6.4-10.3) |  | 57.4 (55.9-58.8) |  | 24.9 (23.1-26.4) |  | 22.6 (20.8-24.4) |  | 21.6 (18.9-24.4) |  |
| **Teaching status** |  | 0.752 |  | 0.007 |  | 0.764 |  | 0.273 |  | 0.835 |
| Non-teaching | 7.8 (6.0-9.9) |  | 56.5 (54.9-57.9) |  | 25.0 (23.1-26.4) |  | 23.2 (21.3-25.0) |  | 20.9 (18.2-23.6) |  |
| Teaching | 8.4 (6.4-10.4) |  | 58.7 (57.1-60.1) |  | 24.9 (23.1-26.2) |  | 22.3 (20.6-24.1) |  | 21.5 (18.8-24.3) |  |
| ***E. coli* isolates** |  |  |  |  |  |  |  |  |  |  |
| **Bed size** |  | 0.032 |  | 0.005 |  | 0.496 |  | 0.384 |  | 0.207 |
| 0-100 | 8.5 (6.0-11.1) |  | 51.1 (49.4-52.9) |  | 28.0 (26.1-29.3) |  | 26.2 (25.3-27.0) |  | 4.4 (3.0-6.4) |  |
| 100-300 | 7.9 (5.4-10.4) |  | 51.7 (49.8-53.5) |  | 28.2 (26.2-29.4) |  | 25.2 (24.3-26.0) |  | 4.5 (3.1-6.6) |  |
| 300 over | 9.5 (6.9-11.9) |  | 52.4 (50.7-54.2) |  | 28.2 (26.3-29.4) |  | 26.3 (25.4-27.1) |  | 4.4 (3.0-6.4) |  |
| **Urban/Rural** |  | 0.067 |  | 0.184 |  | 0.296 |  | 0.379 |  | 0.769 |
| Rural | 8.0 (5.5-10.5) |  | 50.9 (49.0-52.6) |  | 27.7 (25.7-28.9) |  | 25.8 (24.9-26.6) |  | 4.4 (3.0-6.4) |  |
| Urban | 8.8 (6.2-11.3) |  | 52.2 (50.4-54.0) |  | 28.4 (26.5-29.7) |  | 25.8 (24.9-26.6) |  | 4.5 (3.1-6.5) |  |
| **Teaching status** |  | 0.896 |  | 0.003 |  | 0.441 |  | 0.200 |  | 0.496 |
| Non-teaching | 8.4 (5.9-10.9) |  | 50.9 (49.0-52.7) |  | 28.0 (26.1-29.3) |  | 26.1 (25.2-26.9) |  | 4.4 (3.0-6.4) |  |
| Teaching | 8.6 (6.1-11.2) |  | 53.1 (51.4-54.9) |  | 28.4 (26.5-29.6) |  | 25.2 (24.3-26.0) |  | 4.6 (3.2-6.6) |  |
| ***Klebsiella* isolates** |  |  |  |  |  |  |  |  |  |  |
| **Bed size** |  | 0.001 |  | 0.266 |  | 0.015 |  | 0.002 |  | 0.085 |
| 0-100 | 5.3 (4.3-6.2) |  | 82.4 (77.7-87.1) |  | 9.7 (7.6-11.2) |  | 6.3 (5.2-7.4) |  | 53.7 (48.0-59.2) |  |
| 100-300 | 6.4 (5.4-7.5) |  | 88.0 (82.9-92.8) |  | 10.5 (8.6-12.0) |  | 7.2 (6.2-8.3) |  | 57.9 (52.2-63.4) |  |
| 300 over | 8.5 (7.5-9.5) |  | 84.3 (79.5-89.0) |  | 11.6 (9.7-13.0) |  | 5.9 (4.9-7.0) |  | 57.1 (51.4-62.4) |  |
| **Urban/Rural** |  | 0.948 |  | 0.086 |  | 0.475 |  | 0.781 |  | 0.649 |
| Rural | 6.2 (5.2-7.3) |  | 90.1 (84.9-94.8) |  | 10.0 (8.1-11.5) |  | 6.4 (5.3-7.5) |  | 55.1 (49.4-60.5) |  |
| Urban | 7.0 (6.0-8.0) |  | 82.9 (78.1-87.6) |  | 10.9 (8.9-12.5) |  | 6.0 (4.9-7.1) |  | 57.4 (51.6-62.9) |  |
| **Teaching status** |  | 0.366 |  | 0.462 |  | 0.542 |  | 0.686 |  | 0.156 |
| Non-teaching | 6.3 (5.2-7.3) |  | 85.3 (80.1-90.0) |  | 10.3 (8.3-11.9) |  | 6.7 (5.6-7.8) |  | 55.5 (49.8-61.0) |  |
| Teaching | 7.5 (6.6-8.5) |  | 85.6 (80.8-90.3) |  | 11.1 (9.2-12.6) |  | 5.5 (4.4-6.6) |  | 58.3 (52.6-63.8) |  |
| Other Enterobacterales isolates excluding *E. coli* and *Klebsiella* |  |  |  |  |  |  |  |  |  |  |
| **Bed size** |  | 0.381 |  | 0.001 |  | 0.109 |  | 0.430 |  | 0.009 |
| 0-100 | 5.9 (5.7-6.1) |  | 50.8 (50.6-51.1) |  | 21.9 (17.6-26.2) |  | 27.5 (21.9-32.7) |  | 73.3 (70.5-77.6) |  |
| 100-300 | 6.4 (6.2-6.6) |  | 54.5 (54.3-54.8) |  | 25.0 (20.7-29.3) |  | 25.9 (20.2-30.8) |  | 79.0 (76.0-83.1) |  |
| 300 over | 7.1 (6.9-7.3) |  | 57.2 (56.9-57.4) |  | 21.0 (16.7-25.3) |  | 25.3 (19.7-30.3) |  | 74.8 (72.0-78.8) |  |
| **Urban/Rural** |  | 0.621 |  | 0.633 |  | 0.233 |  | 0.023 |  | 0.114 |
| Rural | 6.3 (6.1-6.5) |  | 56.5 (56.3-56.7) |  | 24.8 (20.5-29.1) |  | 29.5 (23.9-34.4) |  | 74.6 (71.6-78.7) |  |
| Urban | 6.6 (6.4-6.8) |  | 52.9 (52.7-53.1) |  | 21.2 (16.9-25.5) |  | 24.4 (18.7-29.6) |  | 76.7 (73.8-80.9) |  |
| **Teaching status** |  | 0.840 |  | 0.275 |  | 0.023 |  | 0.084 |  | 0.321 |
| Non-teaching | 6.3 (6.1-6.5) |  | 54.3 (54.1-54.5) |  | 23.9 (19.6-28.1) |  | 27.5 (21.9-32.7) |  | 76.3 (73.4-80.5) |  |
| Teaching | 6.8 (6.6-7.0) |  | 53.9 (53.7-54.1) |  | 20.1 (15.8-24.4) |  | 23.8 (18.1-28.7) |  | 75.3 (72.5-79.5) |  |

*CI* confidence interval, *ESBL* extended-spectrum beta-lactamase-producing phenotype, *Est* estimated, *NS* not susceptible

**Supplementary Table 4**  Other Enterobacterales organisms excluding *E. coli* and *Klebsiella* spp: adjusted estimates for resistance over time and by geographic region

| **Characteristics** | **ESBL** | | **Beta-lactam** | | **Trimethoprim-sulfamethoxazole** | | **Fluoroquinolone** | | **Nitrofurantoin** | |
| --- | --- | --- | --- | --- | --- | --- | --- | --- | --- | --- |
|  | **Est (95% CI)** | ***P*** | **Est (95% CI)** | ***P*** | **Est (95% CI)** | ***P*** | **Est (95% CI)** | ***P*** | **Est (95% CI)** | ***P*** |
| **Year** |  | 0.799 |  | 0.120 |  | <.001 |  | <0.001 |  | 0.001 |
| 2011 | 6.7 (6.6-6.9) |  | 55.1 (55.0-55.3) |  | 27.0 (26.1-27.8) |  | 32.0 (30.8-33.2) |  | 78.9 (77.9-80.2) |  |
| 2012 | 6.7 (6.6-6.9) |  | 55.1 (54.9-55.2) |  | 25.8 (24.9-26.6) |  | 31.1 (29.9-32.2) |  | 77.3 (76.4-78.6) |  |
| 2013 | 6.6 (6.5-6.8) |  | 54.4 (54.2-54.5) |  | 24.9 (24.0-25.7) |  | 30.1 (28.9-31.2) |  | 76.4 (75.5-77.7) |  |
| 2014 | 6.6 (6.4-6.8) |  | 54.0 (53.8-54.1) |  | 24.8 (23.9-25.7) |  | 29.5 (28.4-30.7) |  | 77.8 (76.9-79.1) |  |
| 2015 | 6.6 (6.4-6.7) |  | 53.8 (53.6-53.9) |  | 23.6 (22.7-24.5) |  | 28.5 (27.1-29.6) |  | 78.3 (77.3-79.6) |  |
| 2016 | 6.6 (6.4-6.7) |  | 54.0 (53.9-54.2) |  | 23.6 (22.5-24.4) |  | 27.1 (26.0-28.3) |  | 75.3 (74.1-76.6) |  |
| 2017 | 6.5 (6.4-6.7) |  | 54.3 (54.2-54.5) |  | 21.5 (20.4-22.5) |  | 25.1 (23.8-26.4) |  | 74.6 (73.5-75.9) |  |
| 2018 | 6.5 (6.3-6.6) |  | 54.3 (54.1-54.5) |  | 21.0 (19.9-22.0) |  | 23.7 (22.6-25.0) |  | 75.3 (74.2-76.6) |  |
| 2019 | 6.4 (6.2-6.6) |  | 53.9 (53.7-54.1) |  | 20.6 (19.7-21.6) |  | 23.4 (22.1-24.7) |  | 74.7 (73.6-76.0) |  |
| 2020 | 6.3 (6.1-6.4) |  | 53.7 (53.5-53.8) |  | 20.1 (19.1-21.0) |  | 22.8 (21.5-24.0) |  | 75.6 (74.4-76.9) |  |
| **Season** (quarter) |  | 0.360 |  | 0.557 |  | <0.001 |  | <0.001 |  | 0.035 |
| 1 | 6.4 (6.3-6.4) |  | 54.3 (54.2-54.4) |  | 23.3 (19.9-26.7) |  | 27.3 (22.8-31.1) |  | 76.3 (74.4-78.9) |  |
| 2 | 6.4 (6.4-6.5) |  | 54.2 (54.2-54.3) |  | 23.0 (19.3-26.4) |  | 26.6 (21.8-30.4) |  | 75.0 (73.0-77.6) |  |
| 3 | 6.5 (6.4-6.6) |  | 54.0 (53.9-54.1) |  | 21.6 (18.2-25.0) |  | 25.0 (20.4-29.4) |  | 75.3 (73.2-78.0) |  |
| 4 | 6.6 (6.5-6.7) |  | 53.9 (53.8-54.0) |  | 22.1 (18.4-25.5) |  | 25.8 (20.9-29.8) |  | 77.2 (75.1-80.0) |  |
| **Census region** |  | 0.027 |  | <0.001 |  | 0.016 |  | <0.001 |  | 0.012 |
| East North Central | 8.4 (7.5-9.6) |  | 57.5 (52.9-61.8) |  | 21.6 (17.3-25.9) |  | 24.9 (19.3-29.9) |  | 77.1 (74.3-81.2) |  |
| East South Central | 6.9 (6.0-8.1) |  | 55.8 (51.3-70.1) |  | 26.2 (21.9-30.5) |  | 31.9 (26.3-36.9) |  | 77.8 (74.8-81.6) |  |
| Middle Atlantic | 6.6 (5.4-7.5) |  | 59.0 (55.4-64.9) |  | 20.3 (16.1-24.6) |  | 26.0 (20.4-30.9) |  | 75.5 (72.6-79.8) |  |
| Mountain | 5.2 (4.4-6.5) |  | 69.5 (66.0-71.9) |  | 22.9 (18.7-27.2) |  | 19.7 (14.1-24.7) |  | 68.0 (65.2-71.9) |  |
| New England | 3.0 (2.3-3.7) |  | 60.2 (56.5-63.4) |  | 14.0 (11.3-17.1) |  | 12.1 (8.5-16.6) |  | 81.1 (79.1-85.1) |  |
| Pacific | 8.2 (7.3-9.4) |  | 59.9 (56.0-63.8) |  | 25.4 (21.2-29.7) |  | 27.3 (21.7-32.2) |  | 71.9 (69.1-75.8) |  |
| South Atlantic | 4.8 (3.6-5.7) |  | 47.0 (43.3-52.9) |  | 18.1 (13.8-22.4) |  | 21.8 (16.2-26.7) |  | 78.6 (75.8-82.6) |  |
| West North Central | 3.4 (2.8-4.5) |  | 52.1 (47.0-54.5) |  | 27.3 (24.4-30.4) |  | 31.2 (27.4-35.5) |  | 84.3 (82.2-88.3) |  |
| West South Central | 5.5 (4.5-6.6) |  | 42.7 (38.6-48.3) |  | 22.3 (18.1-26.6) |  | 25.3 (19.7-30.5) |  | 74.5 (71.7-78.5) |  |

*CI,* confidence interval, *ESBL* extended-spectrum beta-lactamase-producing phenotype, *Est* estimated, *NS* not susceptible
